# Perinatal Mental Health; The Role and the Effect of the Partner: A Systematic Review

**DOI:** 10.3390/healthcare9111572

**Published:** 2021-11-18

**Authors:** Evangelia Antoniou, Pinelopi Stamoulou, Maria-Dalida Tzanoulinou, Eirini Orovou

**Affiliations:** Department of Midwifery, University of West Attica, 12243 Athens, Greece; pstam95@gmail.com (P.S.); dalidatz@gmail.com (M.-D.T.); eorovou@uniwa.gr (E.O.)

**Keywords:** perinatal mental health, perinatal distress, prenatal support, prenatal depression, prenatal anxiety, postnatal depression, postnatal anxiety

## Abstract

Pregnancy is a transitional period involving the most complex experiences in a woman’s life, during which the woman’s psychological status can be affected by a wide range of psychosocial variables. However, positive interpersonal relationships appear to constitute a supportive network that significantly influences perinatal mental health. Therefore, the presence of a supportive partner works psycho-protectively against the difficulties and pressures created by the transition to maternity. The aim of this study was to review systematically the influence of the partner on the woman’s psychology during the perinatal period. Fourteen research articles from PubMed/Medline, Google Scholar and PsycINFO were included in the review from a total of 1846 articles. Most studies have shown a correlation between the support from the partner and prenatal depression and anxiety. Support from the spouse during childbirth is related to the extent to which women feel safe during labor as well as the stress during childbirth. The role of the partner is very important in the occurrence of perinatal mental disorders in women. Of course, more research needs to be done in the field of perinatal mental health. The risk factors that lead to mental disorders need to be clarified and the role of the partner in the perinatal period requires reinforcement and needs to be given the necessary importance.

## 1. Introduction

It is already known that pregnancy and the transition to parenthood include significant biological and psychosocial changes that have been associated with increased anxiety symptoms, depressive symptoms and discomfort. Approximately 20 to 50% of women in developing countries will be confronted with issues related to mental health during the perinatal period [1]. Perinatal mental illness is a major complication of pregnancy and the postpartum period. These disorders include depression, anxiety disorders and postpartum psychosis, which usually manifests as bipolar disorder [2], maternal obsessive compulsive disorder (OCD) [3] and postpartum posttraumatic stress disorder (PTSD) [4]. Perinatal mental illness has been recognized since the time of Hippocrates and commented on through the centuries. The International Marce Society for Perinatal Mental Health, devoted to the study of perinatal mental disorders, has published a significant number of case studies of women suffering from various forms of perinatal mental disorders, 150 years ago [5]. Nowadays, research in this field on the prevalence and risk factors of mental illness has increased dramatically; as a result, many government agencies and specialist teams have developed guidelines for the diagnosis and management of these diseases [6,7,8]. 

Depression and anxiety symptoms have been found to be the most frequent mental health issues that are manifested by women during the perinatal period from the beginning of pregnancy until one year later [9], while proposals have been made to ensure the provision of perinatal mental health services to women two years after childbirth [10]. Depression and anxiety have been found to affect both potential mothers and fathers [11,12]. The manifestation of mental health issues during the perinatal period can be attributed to the drastic psychological and psychosocial changes that the future parents face during the perinatal period [10].

The data presented above suggest that the mental health of both women and men faces many challenges during the perinatal period. However, data deriving from relevant studies regarding the prevalence rates of depression and anxiety symptoms among women and men during the perinatal period is controversial, since the prevalence rates range from 4.8 to 86.5% for depression and from 10.1 to 75.6% for anxiety [13]. Moreover, depression and anxiety symptoms manifested during the perinatal period have been found to have a significantly negative effect on the infant’s mental and physical health, since the manifestation of mental health issues has been found to be positively associated with preterm birth, lower birth weight [14], perinatal effects and cesarean section [15]. Thus, depression and anxiety symptoms during the perinatal period have been found to be both of high prevalence and a significant risk factor for the infant’s health. The symptoms of depression, anxiety and stress that women may experience from conception to one year after giving birth are described as perinatal distress [16]. Although perinatal distress can be connected to physiological processes, the literature shows that the social support [17] and the quality of social relationships are responsible for much of the variability in depressive symptoms among young mothers [18]. 

The term social support refers mainly to any kind of help received from various sources, with an emphasis on how the support is perceived by the recipient [19]. The term “partner support” means the process of responding with supportive actions (behavioral as well as psychological) to a difficulty or problem experienced by the partner in a couple relationship [20]. However, the most significant support source for the reduction of mental health issues during the perinatal period is the relationship with the partner. Many studies indicate that a future mother not only has a wide and effective social support network to deal with the risk of developing mental health problems during the perinatal period [21], but also the future mother’s relationship with her partner has been found to be one of the most effective and important protective factors during the perinatal period [22,23,24,25]. It is a fact that effective supportive behavior from the partner enhances relationship satisfaction and promotes mental and physical health. Satisfaction through a quality relationship, and the intimacy that results from it, has proven to be a strong prognostic factor of adequate mental health in the perinatal period for women [26]. Even in the case of women suffering from depression in pregnancy, the supportive role of the husband managed to minimize these depressive symptoms in the postnatal period [18]. 

Based upon the information provided above, it is evident that the manifestation of depression and anxiety symptoms is of high prevalence during the perinatal period, affecting both women and men. Moreover, the manifestation of mental health issues during the perinatal period is a significant risk factor negatively affecting the infant’s health [27]. Thus, it is significant to investigate potential protective factors that may lower the risk of manifesting mental health issues during this period. Therefore, the aim of this study was to investigate systematically the effect of partner relationship and partner support on the woman’s mental health status during the perinatal period.

## 2. Materials and Methods

The purpose of this study was to investigate the effect of partner relationship and partner support on the mental health status of women during the perinatal period. In particular, we investigated the extent to which parameters such as the couple’s marital status, the quality of their relationship and/or partner support during and after the woman’s pregnancy have an impact on the woman’s mental health status during the perinatal period. In order for this aim to be met, a systematic review of relevant studies was conducted. The research was carried out based on PubMed/Medline, Google Scholar and PsycINFO. 

The keywords used were as follows: Perinatal mental health OR Perinatal distress AND support from spouse OR partner; Perinatal mental health OR Perinatal distress AND the role of partner OR spouse; Perinatal mental health OR Perinatal distress AND partner support OR spouse support; Perinatal mental health OR Perinatal distress AND prenatal depression; Perinatal mental health OR Perinatal distress AND prenatal anxiety; Perinatal mental health OR Perinatal distress AND couple relationship. 

**Figure 1:**
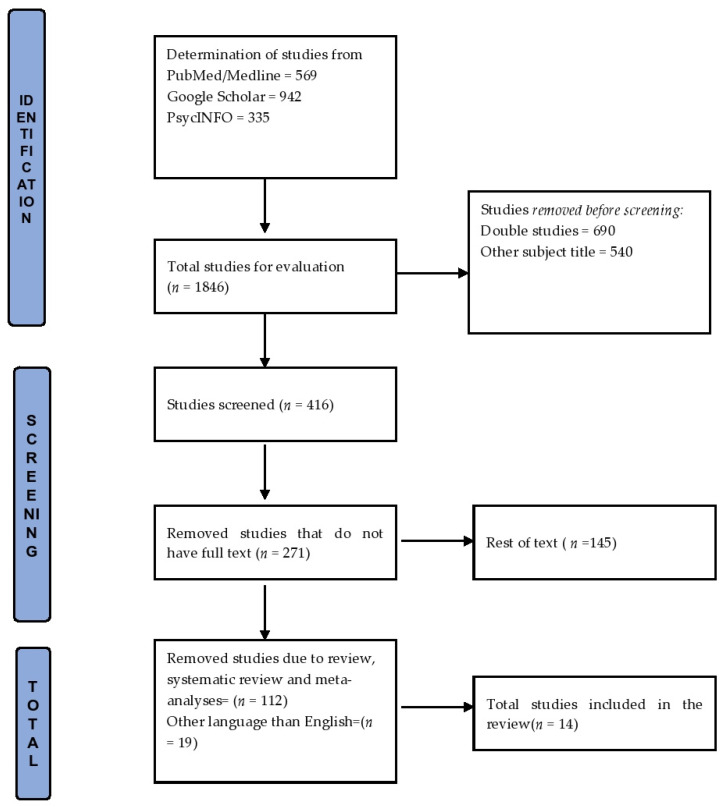
Flow chart: structure search strategy.

The timeline was set from 2010 to 2021 and out of 1846 articles only 14 were included in the review. More specifically, the research articles identified through the initial review were first screened by title and abstract. The full texts of the research papers were examined against the inclusion and exclusion criteria and from a total of 146 studies, a total of 112 reviews, systematic reviews, and meta-analyses were rejected as well as 19 articles in languages other than English (Figure 1). The study analyzed the women’s exposure to low partner support during the perinatal period, as well as domestic violence, infidelity and dissatisfaction with their marriage. As a perinatal outcome, we analyzed the perinatal distress (depression or anxiety or stress) that was documented with specific psychometric tools. 

Regarding the methodological quality of the articles, nine criteria were used to rate them. The first group of criteria (selection) consists of 4 criteria concerning the exposure of the sample. The first criterion, concerning the representative exposure sample, was met by all studies except three. The second criterion concerned the choice of non-exposure, i.e., we did not know before which women were exposed to low support from the partner, was met in all studies. All studies met the third criterion (concerns whether there were exposure findings), because the exposure was identified by the specific psychometric tools. In all studies the outcome did not precede the study (the perinatal distress was the final result of each study), so the fourth criterion was met. The second group of criteria (comparability) consists of 2 criteria concerning adjustment for confusing factors. Therefore, the fifth criterion, which was the adaptation for the educational level, was met in all articles except two. In all studies apart from three, an adaptation for an additional confounding factor had been used. The third group of criteria (results) consists of 3 criteria, evaluating the quality of the results of each study. All studies met the seventh criterion for evaluating the effects of low partner support with psychometric tools and also, in all cohorts and prospective studies there was sufficient follow-up time, thus the cross-sectional studies did not fulfill the eighth criterion. Finally, all articles met non-bias of wear, the ninth criterion. The score of studies varied between 6 and 9 (Table 1).

## 3. Results

The 14 studies included in this review were carried out in various countries. Of the total research, 7 studies were cross-sectional and 7 were prospective or cohort studies (Table 2). Regarding the methodological evaluation of the studies, 6 studies were very good and 7 were of moderate methodological quality (Table 1). More specifically, in the study of Bernard et al. (2018) [28], which was carried out in Jamaica, all available data support a connection link between the low partner support and prenatal depression, with an additional factor of financial difficulties. The mental states of 3517 pregnant women were diagnosed by psychometric tools and the results showed that one in five women had a high likelihood of prenatal depression. The Cheng et al. (2016) [21] cohort study, which analyzed 2 projects, Project Viva and Project ACCESS (the first between 1999 and 2002 and the second between August 2002 and December 2009), highlights higher levels of prenatal anxiety and depression among pregnant women who reported low partner support. Additionally, the women from Project Viva were more likely to smoke, but gained less weight as support from their partners increased. The cohort of Jonsdottir (2017) [29] aimed to investigate the association between the partner relationship and social support when pregnant women are dealing with perinatal distress. However, the results show that pregnant women who were dissatisfied in their partner relationship, mainly with the division of household tasks and child care, were four times more likely to experience perinatal distress, according to the specific psychometric tools. Some other vulnerable factors were lower educational level and unemployment. In addition, the 1062 cases of pregnant women studied by Hartley et al. (2011) [30] were evaluated on the prenatal depressed mood, and the results showed that 39% of women were suffering during pregnancy. The research also shows that low partner support and intimate partner violence constitute the strongest predictors of a depressed mood during pregnancy. Furthermore, two other factors (lower income and younger maternal age) were identified as contributing factors to prenatal distress.

The cross-sectional study of Nasreen et al. (2011) [31] investigated the prevalence of depressive and anxiety symptoms and explored the associated partner involvement. However, the results of this study show that 18% of the sample suffered from depressive symptoms and 29% from anxiety symptoms during pregnancy, associated with physical partner violence and forced sex, as well as the interaction between poor household economy and poor partner relationship. Furthermore, the history of previous depression was an additional factor for prenatal distress. In the cross-sectional study of Yanikkerem et al. (2013) [32], data on 651 pregnant women were collected from a Turkish hospital. In the cases of depressive and anxiety symptoms, the dissatisfaction of women with their marriage is obvious. More specifically, a percentage of 14.5% of pregnant women were more likely to be depressed than women who were satisfied with their marriage. The low educational level of those women and the unplanned pregnancies were additional factors.

The cohort study of Bayrampour et al. (2015) [33] investigated the relationship between prenatal transient and persistent anxiety and risk factors during pregnancy. The results showed that the poor quality of the marital relationship and partner tension were exclusive predictors of anxiety and depressive symptoms in women, in addition to their past psychiatric history.

A recent cross-sectional study of Kazemi et al. (2021) [34] investigated the partner’s emotional reaction to pregnancy. The researchers concluded that the partner’s low emotional reaction to pregnancy was adversely related to prenatal depression and anxiety. Furthermore, the results show that a desirable emotional reaction from the partner decreases anxiety and depression levels that result from an unplanned pregnancy. Another cross-sectional study of Salehi et al. (2017) [35] showed the association between women’s marital satisfaction and anxiety during pregnancy. According to this study, marital satisfaction could predict state anxiety (21%) and trait anxiety (17%) of the pregnant women.

There were 5 studies in our review describing the results of partner support in the postnatal period. The prospective study of Sapkota et al. (2012) [36] examined the impact of prenatal support during labor on maternal anxiety and depressive symptoms in women. The study observations show that continuous support from the spouse during a woman’s labor was associated with a higher degree of postpartum support than for a woman who was not supported by her husband during labor. In the prospective cohort study of Xie et al. (2010) [37], the effect of perinatal family support on postpartum depression was investigated. The results show that low prenatal support from all family members was not associated with increased risk of depression, while low prenatal support from partner and parents was associated with an increased risk. In addition, low postnatal support from all family members was associated with a high risk of depression, suggesting that the support from a partner during the postnatal period was much stronger than the effect of family support before delivery. In the Iles et al. (2011) [38] prospective cohort study, 372 couples were examined for postpartum traumatic stress and depression. The examination took place the first days, six weeks and three months postpartum and the results show that symptoms were significantly related within couples. Moreover, the men’s traumatic symptoms predicted women’s traumatic stress and the women’s dissatisfaction with partner support was associated with greater levels of postnatal depression. In the Stapleton et al. (2012) [39] prospective study, which investigated women’s perceived partner support in pregnancy, a supportive partner may contribute to improved maternal and child well-being after childbirth. In addition, the results show that stronger antenatal partner support predicted lower maternal distress in the postpartum period.

A cross-sectional study on women’s intentions of formal and informal help-seeking in the perinatal period by Fonseca et al. (2017) [40] identified the role of the partner in women’s professional help-seeking and the effect on her mental health problems. More specifically, a significant indirect outcome on the relationship between women’s intention to seek informal and professional help take place through the women’s perceived encouragement from the partner to ask for professional help.

## 4. Discussion

The aim of our systematic review was to describe the effect of partner relationship and partner support on the woman’s mental health status during the perinatal period. The present study provides a strong link between low support from the partner and perinatal distress in women. The findings, according to the included articles, show that the partner’s role is a very important role in the mother’s perinatal period [50]. Actually, for many women, their partner is key in the identification of their perinatal distress and significantly supports the woman in seeking professional help [40,51].

Severe mental health problems during the antenatal period were a more frequent sign in women with low partner support. More specifically, there is a strong correlation between prenatal depression [28,30], prenatal anxiety [33,35] or prenatal distress [21,29,31,32,34], which constitutes an umbrella term to describe anxiety and depressive symptoms during the perinatal period [52], with low partner support. Of course, domestic violence during pregnancy [28,30,31], partner infidelity [28,30] or partner tension [33] are considered factors of low satisfaction in a partner relationship [53]. However, the probabilities that increase the levels of woman’s dissatisfaction in the partner are explained by the positive or negative support provided to her by her partner and financial difficulties. In fact, prosperity in the relationship can be disrupted through financial problems [28,29,30,54], while lower educational levels [29,32], younger age [30] and a history of mental illness [31,33] can be contributing factors to prenatal distress [30]. Furthermore, the fact that women with unplanned and unwanted pregnancies have a significantly higher rate of depression symptoms than women with planned pregnancies [32,34] suggests that they may need more partner and social support.

Certainly, a non-supportive partner during pregnancy will not be able to offer support to the mother during labor and also will not be able to offer her support during the postpartum period [36,55]. This can be explained by the opportunity provided to the partner by his wife to become part of the experience of childbirth and to participate directly in the care of the woman and the infant. At this point, it should be mentioned that a postpartum woman needs more support from her family than in pregnancy, especially from her husband [37]. A possible explanation for this phenomenon could be that some women may be more vulnerable to partner and family postpartum support, due to the need for infant care and the hormonal variability that makes her more sensitive [56].

The high quality and quantity of prenatal support of the partner can contribute not only to the improvement of the health of the mother, but also of the infant during the postpartum period [39]. One possible indirect mechanism is that effective partner support and lower maternal emotional distress could both enable mothers to adopt more sensitive and committed behaviors.

Finally, partner support was a major predictor for maternal distress. A poor-quality couple relationship, particularly in conjunction with other stressors such as financial difficulties, partner infidelity, partner tension and domestic violence, is a strong predictor for perinatal distress. The World Health Organization has advised health policies on mental health problems [57] and especially on maternal mental health [58], emphasizing the importance of early detection and screening for perinatal distress, perinatal care and implications for training.

## 5. Conclusions

Pregnancy is a period of hormonal and emotional changes in a woman’s life. During this period the woman has more access to the health system and therefore, it is a reasonable period to apply screening for prenatal distress. Given the high prevalence of perinatal distress, early midwifery interventions may have important maternal and child outcomes. The role of the partner should be strengthened and included in prenatal care as it seems it is very helpful. The partner’s own mental health should be evaluated at the same time, since it is expected to contribute substantially to the improvement of the mother’s mental health. Furthermore, midwives and other perinatal health care professionals should work together to provide support and empowerment to women and couples throughout the perinatal period. In addition, through the Mental Health Liaison Team, suspicious cases will be detected, evaluated and treated individually.

## Figures and Tables

**Table 1:** Methodological quality of the studies.

Author/Year	Selection	Comparability	Result	Total
1. Bernard (2018) [28]	* * * *	- *	* - *	7
2. Cheng (2016) [21]	* * * *	* *	* * *	9
3. Jonsdottir (2017) [29]	* * * *	* *	* * *	9
4. Hartley (2011) [30]	* * * *	* *	* - *	8
5. Nasreen (2011) [31]	* * * *	* -	* - *	7
6. Yanikkerem (2013) [32]	* * * *	* -	* - *	7
7. Bayrampour (2015) [33]	* * * *	* *	* * *	9
8. Kazemi (2021) [34]	* * * *	* -	* - *	7
9. Salehi (2017) [35]	- * * *	- -	* - *	5
10. Sapkota (2012) [36]	- * * *	* -	* * *	7
11. Xie (2010) [37]	* * * *	* *	* * *	9
12. Iles (2011) [38]	* * * *	* *	* * *	9
13. Stapleton (2012) [39]	* * * *	* *	* * *	9
14. Fonseca (2017) [40]	- * * *	* *	* - *	7

Notes: All 9 criteria were 1. Representative exposure sample, 2. selection of non-exposed, 3. exposure finding, 4. outcome did not precede the study, 5. adaptation for educational level, 6. adaptation for additional confounding factor, 7. outcome evaluation, 8. adequate monitoring time, 9. non-bias of wear. The symbol (*) means that the study met the specific criterion and the symbol; (-) means that the study did not meet it. Selection has 4 criteria 1. 2. 3. 4. Comparability has 2 criteria 5. 6. Results has 3 criteria 7. 8. 9.

**Table 2:** Studies included in the review.

Author/Year	Design	Start/Expiry	N	Data	Country	Exposure	Perinatal Outcomes
1. Bernard, (2018) [28]	Cross-sectional study	1 July–30 September 2011	3517 women	JA-Kids Birth Cohort	Jamaica	Low partner support, violence, partner infidelity	Prenatal depression
2. Cheng (2016) [21]	Cohort study	1999–2009	2641 women	Projects Viva and ACCESS	USA	Low partner support	Prenatal anxiety and depressive symptoms
3. Jonsdottir (2017) [29]	Cohort study	2006–2012	562 women	The Icelandic study of perinatal mental health	Iceland	Dissatisfaction in partner relationship	Prenatal distress
4. Hartley (2011) [30]	Cross-sectional study	2009	1062 women	Philani Mentor Mothers Project	South Africa	Low partner support, intimate partner violence	Prenatal depressed mood
5. Nasreen (2011) [31]	Cross-sectional study	July 2008 to August 2009	720 women	BRAC health program	Bangladesh	Physical partner violence, violence during pregnancy, forced sex, interaction between poor household economy and poor partner relationship.	Prenatal depressive and anxiety symptoms
6.Yanikkerem (2013) [32]	Cross-sectional study	April–September 2011	651 women	Hospital	Turkey	Dissatisfaction with marriage	Prenatal depressive and anxiety symptoms
7. Bayrampour (2015) [33]	Cohort study	2008–2010	3021 women	All Our Babies cohort	Canada	Poor quality of marital relationship, partner tension	Prenatal anxiety
8. Kazemi (2021) [34]	Cross-sectional study	August 2017–April 2018	303 women	Hospitals	Iran	Low partner reaction to pregnancy	Prenatal anxiety and depression
9. Salehi (2017) [35]	Cross-sectional study	March–July 2015	147 women	Health care centers	Iran	Low marital satisfaction	Prenatal anxiety
10. Sapkota (2012) [36]	Prospective study	February–April 2011	231 women	Hospital	Nepal	Lack of support from a husband during labor	Postnatal depression
11.Xie (2010) [37]	Prospective cohort study	February–September 2007	534 women	Maternal and Infant Hospital	China	Low support from partner	Postnatal depression
12. Iles (2011) [38]	Prospective cohort study	Between 2005–2006	372 couples	Hospital	UK	Dissatisfaction with partner support	Postnatal depression
13. Stapleton (2012) [39]	Prospective study	2012 A part from a large survey	272 women	2 large urban medical centers	USA	Low quantity and quality of partner support	Postnatal emotional distress (anxiety and depression
14. Fonseca (2017) [40]	Cross-sectional study	November 2014–March 2015	231 women	Internet survey	Portugal	Less encouragement from partner	Perinatal distress

Notes: The psychometric tools used were as follows: the Edinburgh Postnatal Depression Scale (EPDS) [41], the Beck Depression Inventory (BDI) [42], the State-Trait Anxiety Inventory (STAI) [43], the Social Support Rating Scale (SSRS), the Social Support Effectiveness (SSE) [44], the Significant Others Scale, (SOS) [45], the Enrich marital satisfaction questionnaire, the Hospital Anxiety and Depression Scale (HADS [46], the Adult Attachment Scale (AAS) [47], the Marital Adjustment Test (MAT) [48], the Postpartum Support Questionnaire (PSQ) [49].
